# Air quality and cancer risk in the *All of Us* Research Program

**DOI:** 10.1007/s10552-023-01823-7

**Published:** 2023-12-25

**Authors:** Andrew Craver, Jiajun Luo, Muhammad G. Kibriya, Nina Randorf, Kendall Bahl, Elizabeth Connellan, Johnny Powell, Paul Zakin, Rena R. Jones, Maria Argos, Joyce Ho, Karen Kim, Martha L. Daviglus, Philip Greenland, Habibul Ahsan, Briseis Aschebrook-Kilfoy

**Affiliations:** 1 Institute for Population and Precision Health, University of Chicago, Chicago, IL USA; 2 Department of Public Health Sciences, University of Chicago, Chicago, IL USA; 3 Comprehensive Cancer Center, University of Chicago, Chicago, IL USA; 4 Occupational and Environmental Epidemiology Branch, Division of Cancer Epidemiology and Genetics, National Cancer Institute, Bethesda, MD USA; 5 Division of Epidemiology and Biostatistics, School of Public Health, University of Illinois at Chicago, Chicago, IL USA; 6 Department of Preventive Medicine, Northwestern University Feinberg School of Medicine, Chicago, IL USA; 7 Department of Medicine, University of Chicago, Chicago, IL USA; 8 Institute for Minority Health Research, College of Medicine, University of Illinois at Chicago, Chicago, IL USA

**Keywords:** Cancer risk, PM_2.5_, Air pollution

## Abstract

**Introduction:**

The NIH *All of Us* Research Program has enrolled over 544,000 participants across the US with unprecedented racial/ethnic diversity, offering opportunities to investigate myriad exposures and diseases. This paper aims to investigate the association between PM_2.5_ exposure and cancer risks.

**Materials and methods:**

This work was performed on data from 409,876 *All of Us* Research Program participants using the *All of Us* Researcher Workbench. Cancer case ascertainment was performed using data from electronic health records and the self-reported Personal Medical History questionnaire. PM_2.5_ exposure was retrieved from NASA’s Earth Observing System Data and Information Center and assigned using participants’ 3-digit zip code prefixes. Multivariate logistic regression was used to estimate the odds ratio (OR) and 95% confidence interval (CI). Generalized additive models (GAMs) were used to investigate non-linear relationships.

**Results:**

A total of 33,387 participants and 46,176 prevalent cancer cases were ascertained from participant EHR data, while 20,297 cases were ascertained from self-reported survey data from 18,133 participants; 9,502 cancer cases were captured in both the EHR and survey data. Average PM_2.5_ level from 2007 to 2016 was 8.90 μg/m^3^ (min 2.56, max 15.05). In analysis of cancer cases from EHR, an increased odds for breast cancer (OR 1.17, 95% CI 1.09–1.25), endometrial cancer (OR 1.33, 95% CI 1.09–1.62) and ovarian cancer (OR 1.20, 95% CI 1.01–1.42) in the 4th quartile of exposure compared to the 1st. In GAM, higher PM_2.5_ concentration was associated with increased odds for blood cancer, bone cancer, brain cancer, breast cancer, colon and rectum cancer, endocrine system cancer, lung cancer, pancreatic cancer, prostate cancer, and thyroid cancer.

**Conclusions:**

We found evidence of an association of PM_2.5_ with breast, ovarian, and endometrial cancers. There is little to no prior evidence in the literature on the impact of PM_2.5_ on risk of these cancers, warranting further investigation.

**Supplementary Information:**

The online version contains supplementary material available at 10.1007/s10552-023-01823-7.

## Introduction

Despite decades of improvements in ambient air quality in the United States [1], air pollution remains an environmental exposure of significant interest given disproportionate exposure [2–4] and the impact of even relatively low exposures on health [5] and health outcomes [6–8]. There is an ample evidence of its adverse impact on cardiovascular health [9, 10] and excess mortality [9, 11, 12]. The impact of poor air quality has also been extensively studied for lung cancer [9, 13–17], and associations with cancer have been observed at other organ sites; however, the epidemiological evidence is limited [18–33]. Outdoor air pollution and airborne particulate matter are classified as carcinogenic to humans for lung cancer [31], and evidence points to the need for further investigation of air quality’s impact on cancers including those of the bladder, breast, brain, liver, and kidney [34–36].

The *All of Us* Research Program is enrolling a cohort of over one million participants, offering researchers an unprecedented opportunity to investigate diseases including cancers [37, 38]. Notably, *All of Us* includes participants from racial and ethnic minority groups that have been underrepresented in previous cancer research cohorts [39]. *All of Us* may therefore confer sufficient statistical power to understand the burden of cancer in these populations and identify opportunities for intervention. In the era of precision prevention and precision medicine, investigating the role of the environment in cancer risk is critical [40–42]. Realizing the potential of precision health will call for holistic measures of individual risk that take the physical environment into account.

We recently conducted a preliminary investigation of cancer in the *All of Us* Research Program [43] as part of a demonstration project to show the quality, usefulness, validity, and diversity of the *All of Us* data [44]. We generated descriptive statistics for the most common cancers and considered differences in cancer case ascertainment compared to what would be expected in the broader US population by data source type (self-reported cancer in survey data and/or from the electronic health record). We found that over 13,000 cancer cases were self-reported in the study population of 315,000 people and nearly 24,000 cancer cases were detected in the electronic health records collected for *All of Us* research participants.

Researchers currently have access to data from 409,876 *All of Us* participants through the Researcher Workbench, including residential data for linkage to air pollution exposure. Although the program does not target enrollment by health status, the sample includes sufficient participants with a history of cancer, prevalent cancers, and incident cancers to enable initial investigation of the role of the environment on cancer in the *All of Us* Research Program. Here we investigate the association between ambient air pollution and any health outcome in *All of Us* for the first time, and we present preliminary findings on the association of air quality and cancer in this key precision medicine cohort. We focus on fine particulate matter (PM_2.5_), but our analysis suggests that this is only a first step toward understanding the full impact of diverse environmental factors on cancer and the extensive health outcomes collected by the *All of Us* Research Program.

## Materials and methods

### The *All of Us* Research Program

Data collected from 2017 to 2022 were accessed from the *All of Us* Research Program, a cohort of over 544,000 adults aged 18 and over living in the United States and its territories. The goals, recruitment methods and sites, and scientific rationale for *All of Us* have been described previously [37]. *All of Us* data include participants’ responses to a series of questionnaires, physical measurements collected by study staff at time of enrollment, and information from participants’ Electronic Health Records (EHR). These data are collected either at an *All of Us* affiliated health care provider organization (HPO) or through a “direct-volunteer” mechanism and are made available to researchers via the Researcher Workbench in registered, controlled, and restricted access tiers. Because zip code was required for this analysis, the data for this project were accessed at the controlled tier.

### *All of Us* questionnaire data and physical measurements

Participant-provided information for our analysis including self-reported cancer diagnoses was derived from the *Basics*, *Lifestyle*, and *Personal Medical History* questionnaires. The full text of these questionnaires is available in the Survey Explorer found on the *All of Us* Research Hub, a publicly available website designed to support both researchers and the public [45]. The *Basics* questionnaire elicits demographic information including age, race/ethnicity, education, marital status, household income, and geography. The *Lifestyle* questionnaire collects data on the use of tobacco, alcohol, and other drugs. The *Personal Medical History* questionnaire collects self-reported cancer history. Age at cancer diagnosis in the survey is captured as child (0–11); adolescent (12–17); adult (18–64); older adult (65–74); and elderly (75+). The *Basics* and *Lifestyle* questionnaires are collected at baseline. Until recently, *Personal Medical History* was collected during retention efforts 3 months after enrollment; participants now have the option to complete this questionnaire at the time of enrollment. Body Mass Index (BMI) was calculated using participant height and weight collected by *All of Us* study staff at time of enrollment; height and weight data are housed in the *Physical Measurements* section of the Researcher Workbench.

### EHR-derived cancer diagnoses

Cancer diagnosis data were also derived from participant electronic health records linked to their *All of Us* data. EHR-derived diagnoses were determined using Systematized Nomenclature of Medicine—Clinical Terms (SNOMED CT) codes and mapped to Observational Health and Medicines Outcomes Partnership (OMOP) concept ID by the *All of Us* Data and Research Center. EHR data include procedures, medications, laboratory tests, and health care provider visits. Our analysis used the following OMOP parent concept IDs for cancers/cancer sites: bladder: 93689003, 4095756, 4095755, 197508, 73712, 4312802; blood: 93143009, 109989006, 118601006; bone: 93725000, 78097; brain: 93727008, 4246451; breast: 372137005, 4157332, 4112853; cervix: 372024009, 198984; colon and rectum: 93761005, 36683531, 93984006, 435754, 4180790, 443382, 4180791, 4180792, 443390, 443381, 4181344, 443384; endocrine system: 4241776, 4156115, 371983001; endometrium: 4247238, 4095749; esophagus: 371984007, 4095316, 4094856, 4094854, 4181343, 4089656, 4092060, 4092059, 4094855; eye: 371986009; head, neck, and mouth: 372123001, 372001002, 4090224, 4177101, 4114222, 4089530, 25189, 4178964, 4181350, 4118989, 4090226; kidney: 93849006, 196653, 4091485; lung: 93880001, 443388, 4110587, 254591; ovary: 4116073, 4112864, 93934004, 4181351, 199752; pancreas 372003004, 4092072, 4112734, 4111024, 4178967, 4180793, 4095436; prostate: 93974005, 4163261; stomach: 372014001, 4095320, 4095319, 4149838, 4149837, 4092061, 4095317, 443387l; and thyroid: 94098005, 4178976, 36676291. Year of diagnosis was ascertained for cancer diagnosis, when available. Cases that first appear in the participant EHR after 2006 were included in analysis.

### Air pollution exposure data

Daily PM_2.5_ concentrations were estimated at a resolution of 1 km × 1 km across the contiguous US using a well-validated ensemble-based prediction model that integrates random forest regression, gradient boosting machine, and artificial neural networking [46]. Over 100 variables were used for prediction in this approach including satellite data, land-use information, weather variables, and modeled chemical transport characteristics. We used a 10-year PM_2.5_ average from 2007 to 2016 for our exposure estimate. Output from this approach has been validated with daily PM_2.5_ concentrations measured at 2,156 US EPA monitoring sites. The validation results yielded an average cross-validated *R*-squared value of 0.86 for daily PM_2.5_ predictions, indicating outperformance compared to prior approaches [47, 48].

**Fig. 1:**
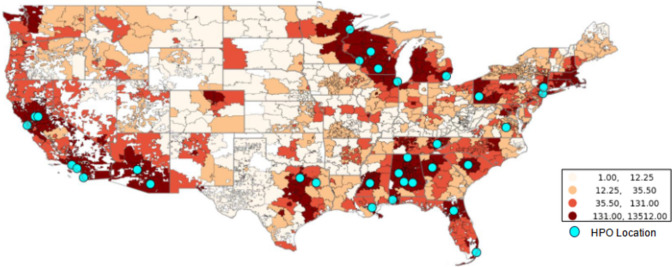
*All of Us* participant population distribution by 3-digit zip code prefix

While residential addresses are not available in the *All of Us* Researcher Workbench, the dataset does contain 3-digit residential zip code prefix for each participant at enrollment. We therefore used zonal statistics to calculate the daily average PM_2.5_ concentration based on all 1 km × 1 km grids within the zip code. Specifically, we identified the 1 km × 1 km grids with centroid in one 3-digit zip code area and then averaged daily PM_2.5_ concentrations across all these grids. The average concentration was thus the PM_2.5_ exposure level for participants in that 3-digit zip code area. Figure 1 shows the distribution of *All of Us* participants represented in this analysis as well as the location of *All of Us* HPO sites.

### Covariates

Following a review of known risk factors for cancer, we selected appropriate variables from the *All of Us* Researcher Workbench data for inclusion in all analyses. Baseline measurements of socioeconomic and demographic covariates including age (19–35, 36–50, 51–65, 65–89), sex at birth (female, male, other), race/ethnicity (non-Hispanic White, non-Hispanic Black, Hispanic/Latino, Asian, other, multiracial, none of the above), current smoking status (yes, no), education (less than high school, high school graduate, some college, college graduate), and BMI (underweight, normal weight, overweight, obese) were included as covariates in the model.

### Data analysis

Data were analyzed in the *All of Us* Researcher Workbench. The Researcher Workbench offers a secure environment and tools to enable users to select cohorts, create datasets for analysis, and conduct analysis using R and Python programming languages in a Jupyter Notebook. We generated descriptive statistics and prevalence for 19 cancers and conducted Chi-square tests to determine the difference in the categorical distribution of data source types (survey data, EHR, and both) across key categories. Descriptive analysis was undertaken on the prevalence of cancer as well as air pollution, and how these were distributed between the different groups of the covariates. To investigate the association between PM_2.5_ and cancer, univariable and multi-variable logistic regression were performed given the rare disease assumption and ability to approximate odds ratio from relative risk for interpretation convenience. Analyses were restricted to cases from the EHR to ensure that the diagnosis date did not occur prior to the exposure. We present the exposure distribution for cases obtained from different sources (EHR, survey, combined) but because date of diagnosis is not available in the survey, we did not include the survey data in logistic models. The first model introduced the unadjusted association between PM_2.5_ exposure and the outcome of interest (cancer overall and by type). The second model was adjusted for age, sex at birth, race/ethnicity, smoking status, education, and BMI. PM_2.5_ concentration was analyzed as a continuous variable as well as categorical variable (quartiles) in the regression models. To evaluate the non-linear relationship between PM_2.5_ exposure and cancer odds, we fitted a generalized additive model (GAM) including a spline term for the accessibility score with 3 degrees of freedom and visualized the exposure–outcome response with adjustment for other covariates. Participants with missing cancer data were excluded and missing values in covariates were treated as an independent category in the analysis. All analyses were conducted using the statistical software R version 4.2.1.

## Results

**Fig. 2:**
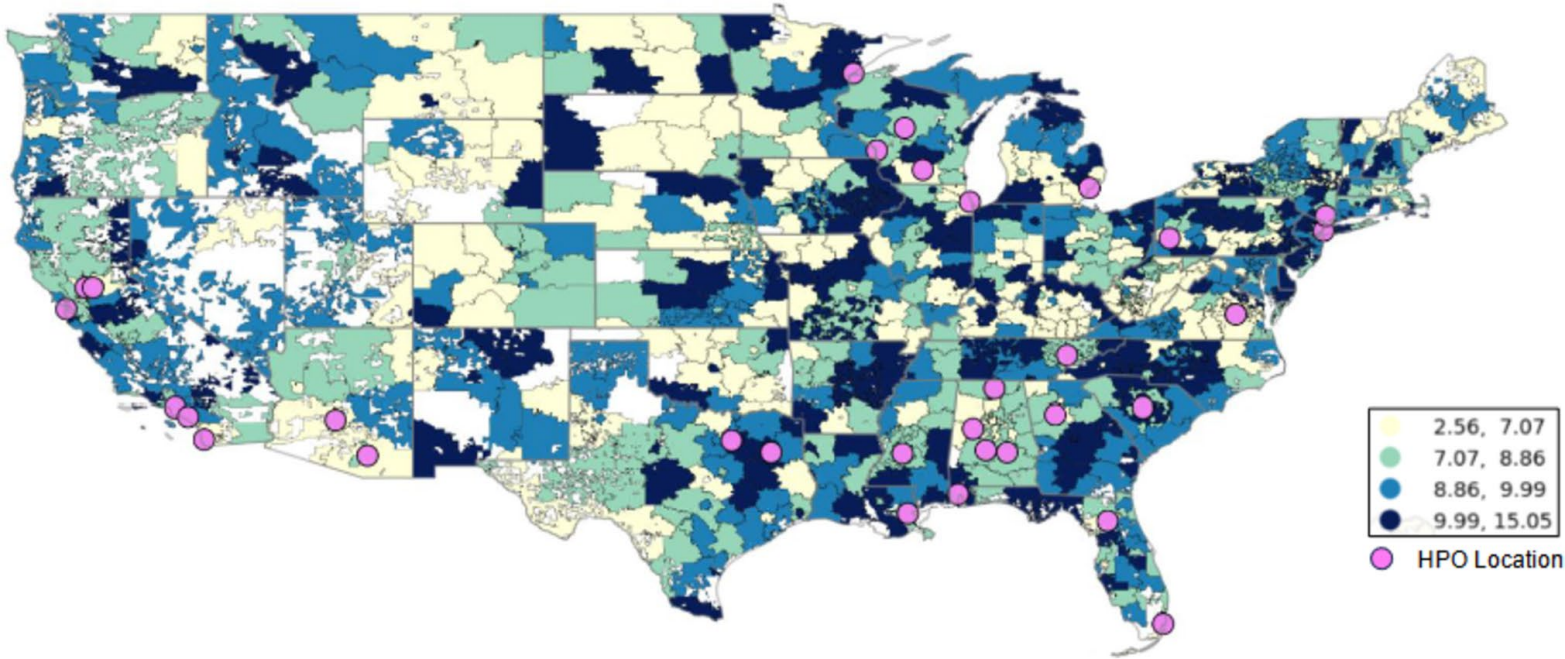
Ambient mean PM_2.5_ estimates in *All of Us* participant locations

Table 1 shows the distribution of the mean annual PM_2.5_ exposure and the baseline characteristics of all participants (*n* = 409,876), among whom 42,462 participants had at least one self-reported or EHR-derived cancer diagnosis. Differences in age, sex at birth, race, smoking status, education, and BMI were observed between the participants overall, with older, female, Non-Hispanic White, non-smoking, more educated, and obese participants more likely to have data on cancer history. We also note differences in cancer outcomes by data source. 33,387 participants had at least one cancer in their EHR (337,292 participants had EHR data), and 18,133 participants reported at least one cancer in the *Personal Medical History* questionnaire (146,815 participants completed this questionnaire). 9,508 participants had at least one cancer in their EHR and in their *Personal Medical History* questionnaire responses. However, mean PM_2.5_ did not vary across these different populations. Figure 2 shows PM_2.5_ levels across the 862 3-digit zip code areas included in this analysis.

**Table 1:** Distribution of *All of Us* Research Program participant characteristics by cancer case ascertainment source

	All participants		Participants with cancer any source (*n* with EHR or survey = 370,965)		Participants with cancer in EHR (*n* with EHR = 337,292)		Participants with cancer in survey (*n* with survey = 180,488)		Participants with cancer in survey and EHR (*n* with EHR and survey = 146,815)	
	*n*	%	*n*	%	*n*	%	*n*	%	*n*	%
Total	409,876	100%	42,462		33,387		18,133		9,508	
Mean PM_2.5_	8.90 μg/m^3^		8.90 μg/m^3^		8.92 μg/m^3^		8.82 μg/m^3^		8.85 μg/m^3^	
Age										
19–35	72,091	17.59%	931	2.19%	738	2.21%	326	1.80%	133	1.40%
36–50	92,790	22.64%	3,822	9.00%	2,698	8.08%	1,602	8.83%	748	7.87%
51–65	119,023	29.04%	11,422	26.90%	9,266	27.75%	4,527	24.97%	2,371	24.94%
66–89	125,972	30.73%	26,287	61.91%	20,865	62.49%	11,678	64.40%	6,256	65.80%
Sex at birth										
Female	247,877	60.48%	24,792	58.39%	19,262	57.69%	11,306	62.35%	5,776	60.75%
Male	153,378	37.42	16,724	39.39%	13,864	41.53%	6,369	35.12%	3,509	36.91%
Other sex	4,497	1.10%	510	1.20%	353	1.06%	299	1.65%	142	1.49%
Missing	4,124	1.01%	436	1.03%	358	1.07%	159	0.88%	81	0.85%
Race/ethnicity										
NH White	219,806	53.63%	29,470	69.40%	22,630	67.78%	14,576	80.38%	7,736	81.36%
NH Black/AA	76,873	18.76%	5,061	11.92%	4,495	13.46%	1,110	6.12%	544	5.72%
Hispanic	73,901	18.03%	4,693	11.05%	4,178	12.51%	1,056	5.82%	541	5.69%
Asian	13,751	3.35%	781	1.84%	633	1.90%	300	1.65%	152	1.60%
Other race	7,107	1.73%	630	1.48%	525	1.57%	216	1.19%	111	1.17%
> 1 race	6,782	1.65%	449	1.06%	338	1.01%	208	1.15%	97	1.02%
Missing	11,656	2.84%	1,378	3.25%	1,038	3.11%	667	3.68%	327	3.44%
Current smoker										
No	320,131	78.10%	37,539	88.41%	29,729	89.04%	16,701	92.10%	8,891	93.51%
Yes	64,875	15.83%	3,963	9.33%	3,300	9.88%	1,134	6.25%	471	4.95%
Missing	24,870	6.07%	960	2.26%	358	1.07%	298	1.64%	146	1.54%
Education										
< High school	36,236	8.84%	2,270	5.35%	2,107	6.31%	318	1.75%	155	1.63%
High school	76,504	18.67%	5,806	13.67%	5,070	15.19%	1,580	8.71%	844	8.88%
Some college	103,390	25.22%	10,609	24.98%	8,485	25.41%	4,314	23.79%	2,190	23.03%
Finished college	180,317	43.99%	22,566	53.14%	17,209	51.54%	11,468	63.24%	6,111	64.27%
Missing	13,429	3.28%	1,211	2.85%	516	1.55%	453	2.50%	208	2.19%
BMI										
Underweight	4,765	1.16%	488	1.15%	418	1.25%	153	0.84%	83	0.87%
Normal weight	86,484	21.10%	9,697	22.84%	8,074	24.18%	4,128	22.77%	2,505	26.35%
Overweight	99,189	24.20%	12,767	30.07%	10,863	32.54%	5,076	27.99%	3,172	33.36%
Obese	134,827	32.89%	15,411	36.29%	13,006	38.96%	5,896	32.52%	3,491	36.72%
Missing	84,611	20.64%	4,099	9.65%	10,206	30.57%	2,880	15.88%	257	2.70%

Table 2 shows that *All of Us* participants’ EHR data indicate a history of breast cancer most frequently (*n* = 8,433; 18.26% of cases) followed by blood cancers (*n* = 5,856; 12.68%), and prostate cancer (*n* = 5,322; 11.53%). More cancers were detected in the EHR passively as opposed to self-reported in the surveys, and the total case numbers are much lower (*n* = 9,502) for cancers cross-referenced in both the EHR and survey data. For the analysis of PM_2.5_ and cancer risk, the case population includes cases detected in the EHR (*n* = 46,176) with a diagnosis date after 2006. The number of cancer cases per participant is summarized in the supplemental table.

**Table 2:** Cancer type distribution by cancer case ascertainment source

	EHR or survey (total cases)		EHR		Survey data		EHR + survey	
	*n*	% dist	*n*	% dist	*n*	% dist	*n*	% dist
Total cancers	56,971		46,176		20,297		9,502	
Bladder	1,808	3.17%	1,432	3.10%	730	3.60%	354	3.73%
Blood	6,784	11.91%	5,856	12.68%	1,704	8.40%	776	8.17%
Bone	2,299	4.04%	2,076	4.50%	328	1.62%	105	1.11%
Brain	1,496	2.63%	1,337	2.90%	287	1.41%	128	1.35%
Breast	11,121	19.52%	8,433	18.26%	5,932	29.23%	3,244	34.14%
Cervix	2,052	3.60%	729	1.58%	1,464	7.21%	141	1.48%
Colon & rectum	3,397	5.96%	2,743	5.94%	1,143	5.63%	489	5.15%
Endocrine system	2,769	4.86%	2,624	5.68%	202	1.0%	57	0.60%
Endometrium	1,349	2.37%	940	2.04%	657	3.24%	248	2.61%
Esophagus	450	0.79%	347	0.75%	164	0.81%	61	0.64%
Eye	493	0.87%	419	0.91%	105	0.52%	31	0.33%
Head, neck, mouth	5,212	9.15%	4,925	10.67%	514	2.53%	227	2.39%
Kidney	2,049	3.60%	1,714	3.71%	760	3.74%	425	4.47%
Lung	3,076	5.40%	2,747	5.95%	736	3.63%	407	4.28%
Ovary	1,468	2.58%	1,162	2.52%	541	2.67%	235	2.47%
Pancreas	925	1.62%	829	1.80%	183	0.90%	87	0.92%
Prostate	6,902	12.11%	5,322	11.53%	3,333	16.42%	1,753	18.45%
Stomach	542	0.95%	448	0.97%	129	0.64%	35	0.37%
Thyroid	2,779	4.88%	2,093	4.53%	1,385	6.82%	699	7.36%

Table 3 presents cancer type distribution across the quartile distribution of PM_2.5_ exposure. More than 25% of blood, brain, breast, cervical, endometrial, and ovarian cancers are observed in the highest exposure quartile (10.67–15.05 µg/m^3^).

**Table 3:** Cancer type distribution by mean annual outdoor PM_2.5_ μg/m^3^ quartile exposure categories

	Total^a^	Q1 (2.56–7.48 μg/m^3^)		Q2 (7.48–9.47 μg/m^3^)		Q3 (9.47–10.67 μg/m^3^)		Q4 (10.67–15.05 μg/m^3^)	
		*n*	%	*n*	%	*n*	%	*n*	%
Overall	46,150	13,185	28.35%	12,703	27.53%	9,113	19.75%	11,149	24.16%
Bladder	1,431	442	30.89%	390	27.25%	286	19.99%	313	21.87%
Blood	5,850	1,626	27.79%	1,490	25.47%	1,152	19.69%	1,582	27.04%
Bone	2,076	710	34.2%	519	25.00%	357	17.2%	490	23.6%
Brain	1,337	393	29.39%	364	27.23%	244	18.25%	336	25.13%
Breast	8,427	2,169	25.74%	2,134	25.32%	1,740	20.65%	2,384	28.29%
Cervix	729	193	26.47%	188	25.79%	143	19.62%	205	28.12%
Colon & rectum	2,742	793	28.92%	924	33.7%	418	15.24%	607	22.14%
Endocrine	2,623	775	29.55%	744	28.36%	538	20.51%	566	21.58%
Endometrium	940	197	20.96%	259	27.55%	223	23.72%	261	27.77%
Esophagus	347	137	39.48%	87	25.07%	58	16.71%	65	18.73%
Eye	418	126	30.14%	122	29.19%	99	23.68%	71	16.99%
Head & neck	4,923	1,355	27.52%	1,528	31.04%	1,064	21.61%	976	19.83%
Kidney	1,713	521	30.41%	458	26.74%	321	18.74%	413	24.11%
Lung	2,746	907	33.03%	732	26.66%	445	16.21%	662	24.11%
Ovary	1,162	299	25.73%	300	25.82%	233	20.05%	330	28.4%
Pancreas	827	272	32.89%	258	31.2%	139	16.81%	158	19.11%
Prostate	5,318	1,520	28.58%	1,517	28.53%	1,122	21.1%	1,159	21.79%
Stomach	448	150	33.48%	116	25.89%	76	16.96%	106	23.66%
Thyroid	2,093	600	28.67%	573	27.38%	455	21.74%	465	22.22%

^a^26 cases were in 3-digit zip code areas with no PM_2.5_ observations

Table 4 reports the odds ratio (OR) and 95% confidence interval (CI) for air pollution with all cancers. The ORs are reported using the first quartile as the reference group. Comparing the highest quartile and lowest quartile of PM_2.5_, strong associations were observed for breast cancer (OR 1.17, 95% CI 1.09–1.25), endometrial cancer (OR 1.33, 95% CI 1.09–1.62), and ovarian cancer (OR 1.20, 95% CI 1.01–1.42). However, some inverse associations were also observed for bone cancer (4th vs.1st quartile: OR 0.78, 95% CI 0.69–0.88); colon and rectum cancer (4th vs. 1st quartile: OR 0.83, 95% CI 0.74–0.93); endocrine system cancer (4th vs. 1st quartile: OR 0.82, 95% CI 0.73–0.92); esophageal cancer (4th vs. 1st quartile: OR 0.55, 95% CI 0.40–0.76); eye cancer (4th vs. 1st quartile: OR 0.70, 95% CI 0.52–0.96), head and neck cancer (4th vs. 1st quartile: OR 0.89. 95% CI 0.82–0.98); lung cancer (4th vs. 1st quartile: OR 0.77, 95% CI 0.69–0.85); pancreatic cancer (4th vs. 1st quartile: OR 0.65, 95% CI 0.52–0.80); prostate cancer (4th vs. 1st quartile: OR 0.85, 95% CI 0.78–0.93); and stomach cancer (4th vs. 1st quartile: OR 0.69, 95% CI 0.53–0.91).

**Table 4:** Cancer odds by increasing quartiles of mean annual PM_2.5_ exposure

	Q1 (2.56–7.48 μg/m^3^)	Q2 (7.48–9.47 μg/m^3^)	Q3 (9.47–10.67 μg/m^3^)	Q4 (10.67–15.05 μg/m^3^)
	OR (95% CI)^a^	OR (95% CI)^a^	OR (95% CI)^a^	OR (95% CI)^a^
Bladder	Ref	0.94 (0.82–1.09)	0.88 (0.75–1.03)	0.91 (0.78–1.07)
Blood	Ref	0.91 (0.84–0.98)	0.82 (0.76–0.89)	1.06 (0.98–1.14)
Bone	Ref	0.71 (0.62–0.80)	0.58 (0.51–0.67)	0.78 (0.69–0.89)
Brain	Ref	0.87 (0.74–1.01)	0.69 (0.58–0.82)	0.93 (0.79–1.09)
Breast	Ref	0.96 (0.90–1.02)	0.83 (0.77–0.89)	1.17 (1.09–1.25)
Cervix	Ref	1.01 (0.81–1.25)	0.80 (0.63–1.02)	0.99 (0.79–1.24)
Colon & rectum	Ref	1.16 (1.05–1.28)	0.61 (0.54–0.69)	0.83 (0.74–0.93)
Endocrine system	Ref	0.94 (0.84–1.04)	0.78 (0.70–0.88)	0.82 (0.73–0.92)
Endometrium	Ref	1.20 (0.98–1.46)	1.11 (0.90–1.36)	1.33 (1.09–1.62)
Esophagus	Ref	0.62 (0.47–0.83)	0.54 (0.39–0.75)	0.55 (0.40–0.76)
Eye	Ref	0.97 (0.75–1.27)	0.99 (0.75–1.30)	0.70 (0.52–0.96)
Head, neck, mouth	Ref	1.11 (1.03–1.20)	0.95 (0.87–1.03)	0.89 (0.82–0.98)
Kidney	Ref	0.92 (0.80–1.05)	0.77 (0.66–0.89)	0.93 (0.81–1.07)
Lung	Ref	0.79 (0.71–0.87)	0.57 (0.50–0.64)	0.77 (0.69–0.85)
Ovary	Ref	0.97 (0.82–1.16)	0.84 (0.70–1.01)	1.20 (1.01–1.42)
Pancreas	Ref	0.99 (0.83–1.19)	0.59 (0.47–0.74)	0.65 (0.52–0.80)
Prostate	Ref	0.96 (0.89–1.04)	0.92 (0.84–1.00)	0.85 (0.78–0.93)
Stomach	Ref	0.75 (0.58–0.97)	0.62 (0.47–0.83)	0.69 (0.53–0.91)
Thyroid	Ref	0.95 (0.84–1.07)	0.87 (0.76–0.99)	0.89 (0.78–1.01)

^a^Adjusted for sex at birth, race/ethnicity, age, smoking status, education, and BMI

Sex and race stratified results are presented in Supplementary Tables 2 and 3. When stratified by sex, blood cancer is significant in males. The race/ethnicity stratified results show increases in blood cancer risk in NH Blacks and Asians as well as significant increases in bone, breast, and endometrial cancers among Hispanics. Asians demonstrated significantly increased risk in pancreatic cancer as well.

**Fig. 3:**
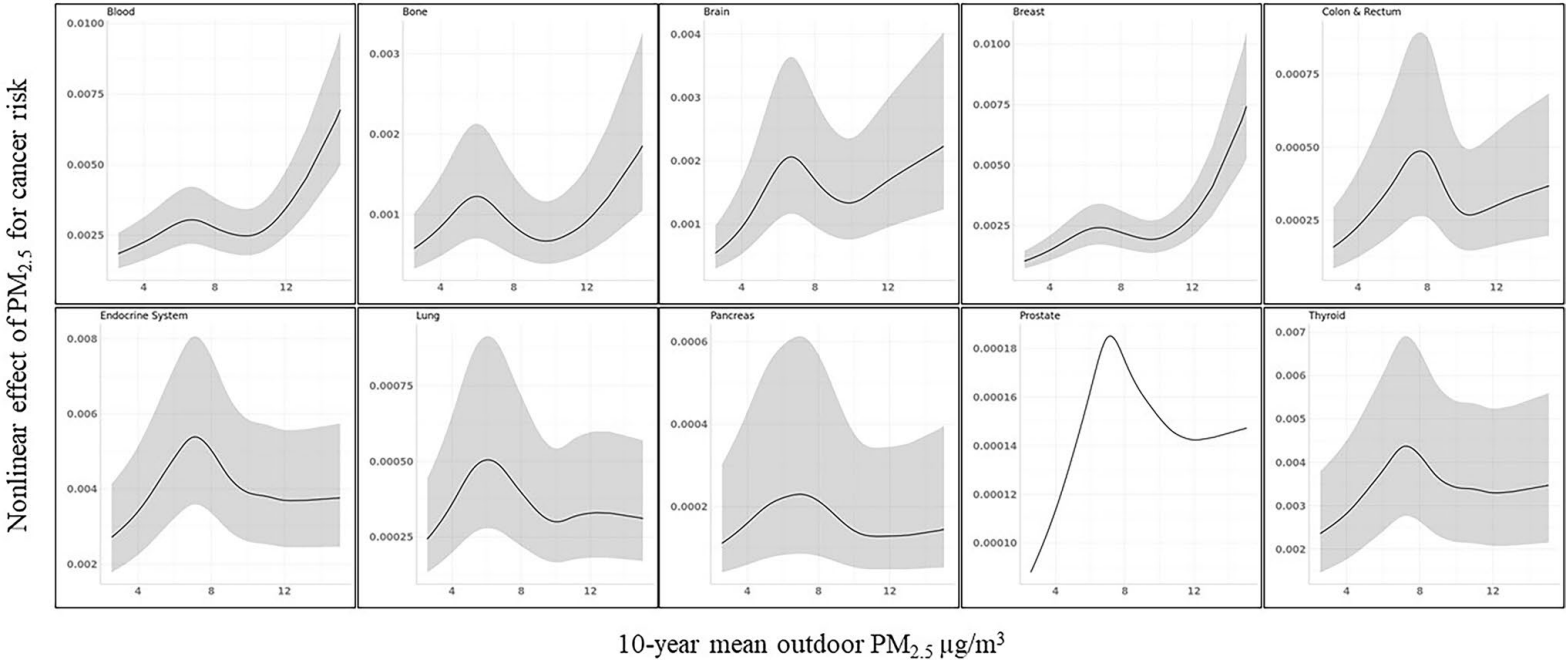
Non-linear relationship between PM_2.5_ and cancer by type

Figure 3 presents the non-linear relationship between PM_2.5_ and cancers with a *p*-value for spline less than 0.10. A non-linear relationship was observed for blood cancer, bone cancer, brain cancer, breast cancer, colon and rectum cancer, endocrine system cancer, lung cancer, pancreatic cancer, prostate cancer, and thyroid cancer. Notably, although we observed inverse associations for bone cancer, lung cancer, and pancreatic cancer in Table 4, results from GAM suggest that high PM_2.5_ concentrations increase the odds for these cancers.

## Discussion

In this study, the mean PM_2.5_ concentration was 8.9 µg/m^3^, in line with the WHO health-based world air-quality guideline [49, 50]. The highest concentration of 15.1 µg/m^3^ was observed in California, while prior review reported an annual average PM_2.5_ concentration of 7.0 µg/m^3^ in the US [33]. The difference can be explained by the spatial distribution of our study population. At present, because urban residents have easier access to *All of Us* HPOs, most participants are concentrated in large cities such as New York City, Chicago, and Los Angeles where the level of air pollution is generally higher than rural areas. However, even the highest PM_2.5_ concentration in this study indicates a recent reduction in average PM_2.5_ exposure level across the US. For instance, a US-wide cohort study based on the American Cancer Society (ACS) Cancer Prevention Study II (CPS-II) reported a median PM_2.5_ concentration of 12.5 µg/m^3^ between 1999 and 2008, with the highest concentration of 28.0 µg/m^3^ [19].

Outdoor air pollution has been classified as Group 1 human carcinogens for lung cancer by the IARC since 2013 [31], a determination based largely on findings from outdoor air pollution exposure analysis in population cohort studies [14, 15]. Similarly, a recent meta-analysis reported a 9% increase in risk for lung cancer incidence or mortality per each 10 µg/m^3^ increase in PM_2.5_ concentration as well as an 8% (95% CI 0–17%) increase in risk per 10 µg/m^3^ for PM_10_ [16]. Our study observed an inverse association between PM_2.5_ and lung cancer. However, this inverse association was only manifest when the exposure level was low, which may reflect measurement error. In our analysis of the variables’ non-linear relationship, the odds for lung cancer increased when PM_2.5_ level exceeded a certain threshold. Therefore, our observation is still consistent with prior conclusions.

While the IARC has reported adverse associations between outdoor air pollution and bladder cancer [31, 51], this association was not observed in our study.

Systemic inflammation, oxidative stress, and epigenetic changes induced by PM exposure [52–55] are thought to play a role in the progression of breast tumors [56–60], and studies from a variety of settings demonstrate an association between PM_2.5_ levels and breast cancer mortality rates as well as all-cause mortality [56, 61]. A recent analysis of 47,433 women in the US Sister Study found adverse associations between PM_2.5_ (HR per 3.6 µg/m^3^, 1.05; 95% CI 0.99–1.11) and breast cancer incidence overall (*n* = 2,848) [22]. An analysis of 57,589 women in the Multiethnic Cohort observed adverse associations of NO_*x*_, NO_2_, PM_2.5_, and PM_10_ and breast cancer incidence among those living within 500 m of major roads [26]. The Canadian National Breast Screening Study (*n* = 89,247) found adverse associations of both PM_2.5_ (HR per 10 µg/m^3^, 1.26; 95% CI 0.99–1.61) and NO_2_ (HRs per 9.7 ppb, range 1.13–1.17) and the risk of incident premenopausal disease [62, 63]. However, no other recent studies have reported clear associations with incident breast cancer risk [23, 64, 65]. In our study, we did observe increased risk for breast cancer associated with PM_2.5_ exposure. This association was more evident when the PM_2.5_ level was high. The finding is generally consistent with previous studies that present suggestive associations for breast cancer. The larger number of breast cancer cases in this study yielded larger statistical power and may explain why we could observe associations in this study.

We also observed significant increased odds for endometrial and ovarian cancers. A recent study conducted in Beijing supports the gynecologic risks associated with air pollution [66]. However, in our study the mean PM_2.5_ concentration was lower than 10 µg/m^3^, a level in line with the World Health Organization (WHO) health-based world air-quality guideline [48, 49]. Our findings warrant further investigation of these cancers in air pollution studies.

A limitation of this study is that we only examined the association of PM_2.5_ with cancers while other pollutants such as SO_2_, NO_2_, NO_*x*_, and O_3_ were not included. PM_2.5_ is the most investigated pollutant and is often used as an indicator of overall air quality. However, the sole investigation of PM_2.5_ may lead to an underestimation of the association between air pollution and cancer risks. For instance, a recent review found that a higher risk of breast cancer was associated with NO_2_ and NO_*x*_, but not PM_2.5_ [60]. Another meta-analysis on leukemia concluded that higher exposure to NO_2_, but not PM_2.5_, was associated with higher leukemia risk. Additionally, this study only includes ambient PM_2.5_ exposure level and relies on historical data. A multi-level approach accounts for multiple pollutants and sources is warranted in future studies.

To preserve participant privacy, the *All of Us* Researcher Workbench only offers participant data at the 3-digit zip code prefix level, rather than at the full 5-digit level which would confer higher spatial resolution for exposure estimates. As the first three digits of a zip code designate a city or a larger rural area, exposure assessment in this study may underestimate geospatial variations in air pollution. Recent epidemiological research has demonstrated the importance of within-city variability in air pollution concentration [67, 68]. However, the current resolution in this study is not sufficient to account for this within-city variability and thus may overlook exposure inequalities faced by urban minorities and underestimate the true associations. Another notable limitation is that we relied on the self-report and electronic health record capture of both incident and prevalent cancers and did not distinguish between primary and secondary cancers. We report differences in the effect based on the source of cancer report. The degree of impact of multiple cancers is illustrated in Supplemental Table 1. Likewise, self-report data are not sufficiently detailed to allow for finer-grained analysis including reproductive or menopausal factors for breast cancer. We also found significant disparity by race in the self-reported survey data. For example, while Non-Hispanic Black participants comprised 18.76% of the overall sample population, they accounted for only 6.12% of self-reported cancers. Similarly, participants identifying as Hispanic/Latino comprised 18.03% of our sample, yet they accounted for only 5.82% of self-reported cancers. This disparity is consistent with our previous analysis of *All of Us* data and highlights the importance of continued engagement with populations historically underrepresented in biomedical research by both incentivizing and removing barriers to follow up data collection [43]. The difference in association between cancer risk and PM_2.5_ based on data source is clearly illustrated in our report. Furthermore, the representativeness of this work is limited given the sampling plan; as illustrated in Figs. 1 and 2, the health provider organizations that account for the greatest share of participant recruitment are generally located in metropolitan areas. Furthermore, at the current stage, the *All of Us* data used for this analysis are cross-sectional in nature as we relied on baseline data and limited longitudinal transfer of EHR. It is therefore difficult to establish temporality between air pollution and cancer outcomes and it is impossible to investigate cancer progression in relation to air pollution. However, reverse causation—the greatest concern in cross-sectional studies—is not likely in this study as higher cancer prevalence does not cause higher air pollution. The association between air pollution and cancer prevalence observed in this study still supports the adverse impact of air pollution on cancer outcomes. Likewise, the cross-sectional nature of the current data also presents the limitation of a lack of “latency” or “lag” of exposure. To address this limitation our analysis used the 10-year PM_2.5_ average from 2007 to 2016, aiming to cover the cancer progression stages before the study enrollment period. However, we understand that these efforts cannot completely offset the limitation induced by the study design. Some inverse associations observed in this study may be the consequence of this limitation.

The study has several notable strengths. While previous studies have been limited by small numbers of cancer cases, the sample size of this study, with more than 400,000 participants, entails the largest investigation of the association between air pollution and cancer to date. Second, research on the carcinogenicity of air pollution has long focused nearly exclusively on lung cancer, however outdoor air pollution might cause cancer at sites other than the lung through absorption, metabolism, and distribution of inhaled carcinogens. Other cancer types, including leukemia and breast cancer, have been also investigated in relation to air pollution. However, to our knowledge no study has simultaneously investigated as many cancer types as in this one. Third, the study design of *All of Us* will eventually enable researchers to analyze cancer risk longitudinally (although in this early analysis we are restricted to essentially cross-sectional data), thus providing additional opportunities to consider the role of air pollution in cancer occurrence and development. Many prior studies have only been able to use cancer mortality as the outcome, thus may underestimate the true odds for some cancers.

In summary, the *All of Us* Research Program presents significant opportunities to further evaluate the role of the environment and air pollution in cancer odds and outcomes. We have observed associations of PM_2.5_ exposure with several types of cancer and risks differing by race/ethnicity. This preliminary investigation suggests that some previous findings on cancer and PM_2.5_ are also observed in *All of Us*; for instance, our breast cancer results. Given the large and diverse *All of Us* study population, it may be possible to further consider the role of the environment on cancer disparities in addition to cancer risk in general. In the coming years, *All of Us* may confer sufficient study power to research the role of the environment in cancers that have historically been infeasible to investigate due to small sample size. This project should provide some preliminary insight and direction for future investigation.

### Supplementary Information

Below is the link to the electronic supplementary material.

Supplementary file1 (DOCX 20 kb)

## Data Availability

Data are owned by a third party, the *All of Us* Research Program. The data underlying this article were provided by the *All of Us* Research Program by permission. Data will be shared on request to the corresponding author with permission of *All of Us*. More information on data access can be found in the *All of Us* Research Hub (https://www.researchallofus.org).
