# Supplementary material for: Air quality and cancer risk in the All of Us Research Program
Source: Cancer Causes Control. 2023 Dec 25;35(5):749–60. doi: 10.1007/s10552-023-01823-7 (PMC11045436; doi:10.1007/s10552-023-01823-7)
Supplement: Supplementary file 1 — Supplementary file1 (DOCX 20 kb) [file 10552_2023_1823_MOESM1_ESM.docx]

**Supplementary Table 1** Number of cancer types reported per participant in EHR and survey

| **# Reported cancer types per participant** | **Total EHR cancers** | **Total survey data cancers** |
| --- | --- | --- |
| Total | 46,176 | 18,133 |
| 1 | 25,014 | 16269 |
| 2 | 6,361 | 1613 |
| 3 | 1,693 | 217 |
| 4 | 565 | 24 |
| 5 | 154 | 8 |
| 6 | 38 | 1 |
| 7 | 10 | 1 |
| 8 | 3 | 0 |
| 9 | 1 | 0 |

**Supplementary Table 2** Overall and sex-stratified results by increasing unit of PM_2.5_

|  | **Overall** | **Female** | **Male** |
| --- | --- | --- | --- |
|  | OR (95% CI) | OR (95% CI) | OR (95% CI) |
| Bladder | 0.98 (0.97-1.02) | 0.99 (0.95-1.04) | 0.99 (0.96-1.02) |
| Blood | 1.02 (1.00-1.03) | 1.01 (0.99-1.03) | 1.02 (1.00-1.04) |
| Bone | 0.95 (0.93-0.97) | 0.95 (0.92-0.98) | 0.95 (0.93-0.98) |
| Brain | 0.99 (0.97-1.02) | 0.99 (0.96-1.03) | 0.99 (0.95-1.03) |
| Breast | 1.03 (1.02-1.04) | 1.03 (1.02-1.04) | ~ |
| Cervix | 0.99 (0.96-1.03) | 0.99 (0.96-1.03) | ~ |
| Colon & Rectum | 0.96 (0.95-0.98) | 0.96 (0.94-0.99) | 0.96 (0.94-0.99) |
| Endocrine System | 0.97 (0.96-0.99) | 0.97 (0.95-0.99) | 0.97 (0.94-1.01) |
| Endometrium | 1.06 (1.03-1.10) | 1.06 (1.03-1.10) | ~ |
| Esophagus | 0.90 (0.85-0.94) | 0.92 (0.84-1.00) | 0.89 (0.84-0.99) |
| Eye | 0.99 (0.51-1.04) | 1.02 (0.99-1.04) | 0.98 (092-1.04) |
| Head & Neck | 0.99 (0.98-1.01) | 1.01 (0.99-1.03) | 0.99 (0.97-1.01) |
| Kidney | 0.97 (0.95-0.99) | 0.97 (0.94-1.01) | 0.97 (0.94-1.01) |
| Lung | 0.94 (0.93-0.96) | 0.95 (0.92-0.97) | 0.94 (0.92-0.97) |
| Ovary | 1.02 (0.99-1.05) | 1.02 (0.99-1.05) | ~ |
| Pancreas | 0.93 (0.90-0.96) | 0.94 (0.90-0.99) | 0.92 (0.87-0.96) |
| Prostate | 0.99 (0.98-1.01) | ~ | 0.99 (0.98-1.01) |
| Stomach | 0.94 (0.90-0.99) | 0.98 (0.92-1.04) | 0.91 (0.86-0.97) |
| Thyroid | 0.99 (0.97-1.01) | 0.99 (0.96-1.01) | 0.99 (0.95-1.03) |

* adjusted for sex at birth, race/ethnicity, age, smoking status, education, and BMI

Supplementary Table 3 Race/ethnicity-stratified results by increasing unit of PM_2.5_

|  | **NH White** | **NH Black/AA** | **Hispanic** | **Asian** |
| --- | --- | --- | --- | --- |
|  | OR (95% CI) | OR (95% CI) | OR (95% CI) | OR (95% CI) |
| Bladder | 1.04 (0.78-1.39) | 0.95 (0.85-1.06) | 1.05 (0.96-1.14) | 1.04 (0.78-1.39) |
| Blood | 1.18 (1.05-1.33) | 1.06 (1.02-1.11) | 1.00 (0.97-1.04) | 1.18 (1.05-1.33) |
| Bone | 1.06 (0.91-1.23) | 1.04 (0.96-1.13) | 1.08 (1.02-1.15) | 1.06 (0.91-1.23) |
| Brain | 1.04 (0.87-1.25) | 1.08 (0.98-1.20) | 1.05 (0.98-1.12) | 1.04 (0.87-1.25) |
| Breast | 1.03 (1.02-1.04) | 1.00 (0.96-1.04) | 1.04 (1.01-1.07) | 1.01 (0.94-1.09) |
| Cervix | 1.01 (0.96-1.07) | 0.92 (0.84-1.02) | 1.01 (0.94-1.09) | 0.90 (0.70-1.15) |
| Colon & Rectum | 0.99 (0.86-1.15) | 0.97 (0.91-1.04) | 1.00 (0.96-1.05) | 0.99 (0.86-1.15) |
| Endocrine System | 1.02 (0.89-1.16) | 0.97 (0.90-1.04) | 0.97 (0.93-1.01) | 1.02 (0.90-1.16) |
| Endometrium | 1.08 (1.03-1.12) | 0.93 (0.83-1.03) | 1.10 (1.02-1.19) | 1.05 (0.77-1.42) |
| Esophagus | 0.83 (0.56-1.25) | 0.88 (0.72-1.07) | 0.87 (0.73-1.04) | 0.83 (0.56-1.25) |
| Eye | 0.89 (0.57-1.39) | 1.20 (0.85-1.69) | 0.94 (0.80-1.11) | 0.89 (0.57-1.39) |
| Head & Neck | 0.86 (0.72-1.03) | 1.02 (0.93-1.11) | 0.98 (0.93-1.03) | 0.86 (0.72-1.03) |
| Kidney | 1.34 (0.98-1.84) | 1.01 (0.94-1.10) | 0.95 (0.90-1.00) | 1.34 (0.98-1.84) |
| Lung | 1.00 (0.88-1.15) | 1.00 (0.94-1.07) | 0.96 (0.91-1.01) | 1.00 (0.88-1.15) |
| Ovary | 1.02 (0.98-1.05) | 0.89 (0.80-0.99) | 1.04 (0.97-1.11) | 1.22 (0.98-1.52) |
| Pancreas | 1.35 (1.03-1.78) | 0.89 (0.80-0.99) | 1.05 (0.96-1.14) | 1.35 (1.03-1.78) |
| Prostate | 1.00 (0.98-1.02) | 0.96 (0.92-1.01) | 0.97 (0.93-1.01) | 0.93 (0.81-1.08) |
| Stomach | 0.97 (0.69-1.36) | 0.93 (0.81-1.07) | 1.08 (0.97-1.20) | 0.97 (0.69-1.36) |
| Thyroid | 1.02 (0.89-1.17) | 0.98 (0.90-1.07) | 0.95 (0.91-0.99) | 1.02 (0.89-1.17) |

* adjusted for sex at birth, race/ethnicity, age, smoking status, education, and BMI
